# *Bacillus coagulans* MTCC 5856 supplementation in the management of diarrhea predominant Irritable Bowel Syndrome: a double blind randomized placebo controlled pilot clinical study

**DOI:** 10.1186/s12937-016-0140-6

**Published:** 2016-02-27

**Authors:** Muhammed Majeed, Kalyanam Nagabhushanam, Sankaran Natarajan, Arumugam Sivakumar, Furqan Ali, Anurag Pande, Shaheen Majeed, Suresh Kumar Karri

**Affiliations:** 1 Sami Labs Limited, Peenya Industrial Area, Bangalore, 560 058 Karnataka India; 2 Sabinsa Corporation, 20 Lake Drive, East Windsor, NJ 08520 USA; 3 Sabinsa Corporation, 750 Innovation Circle, Payson, UT 84651 USA; 4 ClinWorld Private Limited, # 19/1 & 19/2, I Main, II Phase, Peenya Industrial Area, Bangalore, 560 058 Karnataka India

**Keywords:** Lactobacillus, *Bacillus coagulans*, LactoSpore®, Diarrhea, Irritable Bowel Syndrome

## Abstract

**Background:**

*Bacillus coagulans* MTCC 5856 has been marketed as a dietary ingredient, but its efficacy in diarrhea predominant irritable bowel syndrome (IBS) condition has not been clinically elucidated till date. Thus, a double blind placebo controlled multi-centered trial was planned to evaluate the safety and efficacy of *B. coagulans* MTCC 5856 in diarrhea predominant IBS patients.

**Methods:**

Thirty six newly diagnosed diarrhea predominant IBS patients were enrolled in three clinical centres. Along with standard care of treatment, 18 patients in group one received placebo while in group two 18 patients received *B. coagulans* MTCC 5856 tablet containing 2 × 10^9^ cfu/day as active for 90 days. Clinical symptoms of IBS were considered as primary end point measures and were evaluated through questionnaires. The visual analog scale (VAS) was used for abdominal pain. Physician’s global assessment and IBS quality of life were considered as secondary efficacy measures and were monitored through questionnaires.

**Results:**

Laboratory parameters, anthropometric and vital signs were within the normal clinical range during the 90 days of supplementation in placebo and *B. coagulans* MTCC 5856 group. There was a significant decrease in the clinical symptoms like bloating, vomiting, diarrhea, abdominal pain and stool frequency in a patient group receiving *B. coagulans* MTCC 5856 when compared to placebo group (*p* < 0.01). Similarly, disease severity also decreased and the quality of life increased in the patient group receiving *B. coagulans* MTCC 5856 when compared to placebo group.

**Conclusions:**

The study concluded that the *B. coagulans* MTCC 5856 at a dose of 2 × 10^9^ cfu/day along with standard care of treatment was found to be safe and effective in diarrhea predominant IBS patients for 90 days of supplementation. Hence, *B. coagulans* MTCC 5856 could be a potential agent in the management of diarrhea predominant IBS patients.

## Background

The World Health Organization in 2001 defined probiotics as "live micro-organisms which, when administered in adequate amounts, confer a health benefit on the host" [1] and are able to prevent or improve some disease conditions. Consumption of probiotics is associated with a range of health benefits including stimulation of the immune system, protection against diarrheal diseases, nosocomial and respiratory tract infections, lowering of cholesterol, attenuation of overt immuno inflammatory disorders and anticancer effects [2, 3]. Most Probiotic microorganisms belong to the genera *Lactobacillus* and *Bifidobacterium*; however, other bacteria and some yeast may also possess probiotic properties. Lactobacilli are usually described as Gram-positive, non-spore-forming and non-flagelated rods or cocobacilli, aerotolerant, fastidious, acid-tolerant, and strictly fermentative. A recent study revealed that the probiotic *Bacillus coagulans* MTCC 5856 in combination with an aqueous extract of cinnamon has strong synergetic effects on phagocytosis and on regulation of cholesterol and blood sugar levels and also confirmed that the combination reduced intestinal damage in mouse model of colitis [4].

The commercial interest in functional foods containing probiotics strains has consistently increased due to the awareness of the benefits for gut health and disease prevention and therapy [5]. Some probiotics have been shown in preliminary research to possibly treat various forms of gastroenteritis [6]. It is important to note that health benefits provided by probiotics are strain specific, and not species or genus-specific. Therefore, it should be noted that no probiotic strain will provide all proposed benefits, not even strains of the same species, and not all strains of the same species will be effective against defined health conditions [7–10]. The results of genotypic sequencing indicated that the *B. coagulans* MTCC 5856, the probiotic strain under study, showed more than 99 % similarity with *B. coagulans* ATCC 31284 (1048/1050; differed in only 2 of 1050 base pairs), *B. coagulans* ATCC 7050 (1049/1050; differed in only 1 of 1050 base pairs) and *B. coagulans* NBRC 3887 (1049/1050; differed in only 1 of 1050 base pairs). Thus, *B. coagulans* MTCC 5856 shared more than 99 % 16S rDNA sequence homology with *B. coagulans* NBRC 3887, *B. coagulans* ATCC 31284 and *B. coagulans* ATCC 7050 but differed in few base pairs (Majeed et al. Unpublished data). This was an indication that different strains of the same species may have different phenotypic and genotypic profile. Therefore, the evaluation of *B. coagulans* MTCC 5856 efficacy in IBS patients is essential.

Probiotic bacteria *B. coagulans* MTCC 5856 has been in the market as a dietary ingredient for nearly two decades, under the trade name LactoSpore®. *B. coagulans* MTCC 5856 is a room temperature stable, lactose free and non-GMO probiotic preparation with GRAS status. The strain MTCC 5856 has the ability to withstand high heat and has been included in functional baked foods [11]. Recent study suggested that the *B. coagulans* MTCC 5856 did not alter either genetically or phenotypically and was found to be consistent over multiple years of commercial production [12]. However, the safety of *B. coagulans* MTCC 5856 has not been adequately established in diarrhea predominant IBS patients. Thus, the current double-blind, placebo-controlled, multi-centered, two arm study was conducted to evaluate *B. coagulans* MTCC 5856 safety and efficacy as dietary supplement in patients receiving standard care of treatment for diarrhea predominant IBS patients.

## Methods

### Tablet formulation

The active product *B. coagulans* MTCC 5856, 2 billion spores per tablet (2 × 10^9^ spore/tablet), was supplied by Sabinsa Corporation, Utah, USA. Tablets were packed in 70 mL HDPE container. Each active had 2 billion spores per tablet i.e., 333.33 mg of *B. coagulans* MTCC 5856, 222.67 mg of microcrystalline cellulose, 10 mg of starch, 30 mg of sodium starch glycolate and 4.0 mg of magnesium stearate. Viable spore count of *B. coagulans* MTCC 5856 was determined as per the method described previously [13] Briefly, 1.0 g of *B. coagulans* MTCC 5856 was mixed in sterile saline (0.9 % NaCl, w/v) and then incubated in a water bath for 30 min at 75 °C, followed by immediate cooling to below 45 °C. The suspension was further serially diluted in sterile saline and the viable count was enumerated by plating on glucose yeast extract agar (HiMedia, Mumbai, India) by pour plate method. The plates were incubated at 37 °C for 48–72 h. Analysis was performed twice in triplicate. Average means of spore viable counts was expressed in cfu/g. For the placebo tablet, maltodextrin of equivalent weight was used and formulated in similar shape and size as that of the active, and packed in HDPE containers.

### Ethics and informed consent

The trial (Clinical Trial Registry India, # CTRI/2014/03/004502) was conducted in three clinical sites i) Mysore Medical College and K R Hospital, Mysore, India ii) Sapthagiri Institute of Medical Sciences and Research Center, Bangalore, India and iii) Kempegowda Institute of Medical Sciences, Bangalore, India. The institutional ethics committees of the aforesaid clinical sites provided a written favorable opinion for the conduct of this study in respective clinical sites. No further changes or amendments were made to the approved protocol and the study was executed in its complete form. This trial was conducted in accordance with the principles enunciated in the Declaration of Helsinki (Edinburgh, 2000) [14] and the ICH-harmonized tripartite guideline regarding good clinical practice (GCP). Written and oral information about the study was provided to all the subjects in a language understandable by the subject. Every subject was informed by the investigator, prior to the screening evaluation, of the purpose of this clinical trial, including possible risks and benefits and documented the informed consent process in the subject’s chart. Sufficient time was provided for each subject to decide whether to participate in the study and all the questions and clarifications regarding the study were clarified by the investigator.

### Study design and selection of study subjects

This randomized, double blind, parallel group, placebo controlled, multi-centered study had a total of 5 visits to the clinical site by the study subjects, besides screening visit. Subjects were included in the study if indicated “Yes” to all of the inclusion criteria and “No” to any of the exclusion criteria. **Inclusion Criteria:** 1) Male or female subjects ranging in age from 18 to 55 years (both inclusive) diagnosed as having gastro intestinal disorders and based on the medical history record were included in the study by the investigator. 2) Fulfilling Rome III diagnostic criteria for functional IBS [15]. Criterion fulfilled for the last 3 months with symptom onset at least 6 months prior to diagnosis. a) Recurrent abdominal pain or discomfort (uncomfortable sensation not described as pain) at least 3 days/month in the last 3 months associated with two or more of the following: (i). Improvement with defecation (ii). Onset associated with a change in frequency of stool (iii). Onset associated with a change in form (appearance) of stool. b) Recurrent feeling of bloating or visible distension at least 3 days/month in the last 3 months. c) Loose (mushy) or watery stools without pain occurring in at least 75 % of stools. 3) Willingness to follow the protocol requirement as evidenced by written, informed consent. 4) Willingness to complete subject diaries and respond to study questionnaires. 5) Except standard treatment of care, agree not to use any other (including vitamins and minerals) medication during the course of the study. 6) Agree not to use any yogurt during the course of this study. 7) Subjects whose blood chemistries are within a normal range or not considered clinically significant if outside the normal range. 8) Subject’s assurance that they have not taken antibiotics or other products whose primary site of action is in the gastro intestinal tract (GIT) for a period up to 1 month prior to the start of the study. **Exclusion Criteria:** 1) Sufficient criteria for a diagnosis of functional dyspepsia or other functional GI disorder. 2) Any clinically significant medical history, medical finding or an ongoing medical or psychiatric condition exists which in the opinion of the Investigator could jeopardize the safety of the subject, impact validity of the study results or interfere with the completion of study according to the protocol. 3) Significant abnormal findings as determined by baseline history, physical examination, vital signs (blood pressure, pulse rate, respiration rate) hematology, serum chemistry, urinalysis. 4) History or presence of significant alcoholism or product abuse in the past one year. 5) Participation in a clinical study during the preceding 90 days. 6) History of malignancy or other serious disease. 7) Any contraindication to blood sampling. 8) Smoking or consumption of tobacco products. 9) Blood or blood products donated in past 30 days prior to study ingredient administration. 10) Female subjects on pregnancy and lactating women.

### Sample size calculations, randomization and treatment allocation and procedures

A sample size of 30 subjects was calculated based on a 40 % reduction in severity of clinical symptoms of IBS (bloating, vomiting, diarrhea, abdominal pain, stool frequency) between the two treatment groups with a power of 80 % at the 5 % level of statistical significance. With an expected dropout rate of 20 %, the sample size was increased to 36 (18 subjects per group). Eligible subjects were randomized in a 1:1 ratio (placebo: active) in a randomly-permuted order by computer into 3 blocks of 16 each, along with overages. Each participant was assigned a 6-digit randomization code and the respective site personnel dispensed the investigational product as per the randomization code list generated by an independent statistician. Clinical site staff and participants remained blinded to the treatment received throughout the course of the study. Double blinding was accomplished by independent blinding of the dosing kits. Newly diagnosed or untreated patients who were not on any other treatment in the past 3 months with mild to moderate IBS in severity were enrolled into the study. Two drugs, one a combination of Domperidone 30 mg and Esomeprazole 40 mg, and the other drug Metronidazole 400 mg, once a day was considered as standard treatment of care for diarrhea predominant IBS for the study subjects for both active and placebo groups, by the investigators of the three clinical sites. In addition to the aforesaid standard treatment, subjects were asked to self administer one tablet per day, either placebo or active, at least 30 min before a meal, preferably in the morning as a dietary supplement for a period of 90 days. This was subject to a gap of at least 4 h between the study product (placebo/active) and standard care of treatment was maintained. Subjects used this product on an outpatient basis and were advised to return for clinical evaluation at day 30, day 60, day 90 and day 105. The dosing period was for 90 days.

**Fig. 1:**
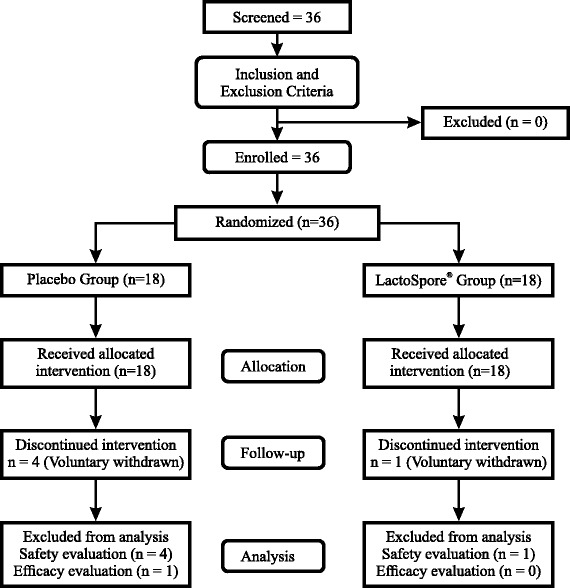
Flow chart of study procedures. Legend: From eligible thirty six subjects who were fit into the inclusion and exclusion criteria, 31 completed the study. At every follow up visit, study evaluations and assessments were made in both the study groups

Compliance with study supplement was reviewed at each visit by examination of the returned supplements. All accountability records were incorporated into the investigator’s study file. The patients were instructed against the use of any kind of yoghurt during the study duration. The daily food intake of the patients was recorded in the patient diaries provided to them at visit 1. The same was verified at subsequent visits by the investigators. Respective hospital laboratories were used for all assessments pertaining to this study. Clinical trial monitors who were independent of the study staff monitored the progress of all clinical investigations that were conducted and ensured that the protocol is adhered in all aspects. Data collection during this clinical study and statistical analysis were performed by separate functional groups and a certified, independent statistician respectively. No changes or amendments were made to the approved protocol after the trial commenced and no interim analysis was done during the study period. The screening and enrollment of study subjects is seen in Fig. 1.

### Safety and efficacy outcomes

The safety outcomes were measured by: 1) Physical examination and vitals, 2) Assessment of reported adverse events (AEs), if any. The primary efficacy outcomes were measured by 1) modified gastro intestinal (GI) discomfort questionnaire for bloating, vomiting and diarrhea [16] 2) Stool frequency and consistency by subjective evaluation using Bristol stool form score [17]. 3) Self assessment of abdominal pain, measured on a 10 cm visual analog scale –VAS [18], 2) The secondary efficacy outcomes were measured by 1) Physician’s global assessment for disease severity [19], 2) A 34 item IBS quality of life (QOL) questionnaire [20].

### Statistical analysis

The baseline values of VAS, GI discomfort questionnaire, Bristol stool form score, Physician’s global assessment and IBS QOL questionnaire were compared to that of end of study visit for both treatment groups by appropriate statistical tools. Statistical analysis software (SAS) version 9.2 software was used for data analysis. Paired ‘*t*’ test, analysis of covariance (ANCOVA) and Wilcoxon signed rank sum test were used for appropriate data set variables to reach the best possible statistical conclusion between the active and placebo receiving groups. A ‘*p*’ value <0.05 was considered as statistically significant. The baseline descriptors were summarized as mean and standard deviation for continuous variables and as frequencies and percentages for categorical variables. Last observation carry forward (LOCF), the intent to treat method was followed for efficacy evaluations of subjects.

## Results

The first patient was enrolled in March 2014 and the last subject completed the study in July 2014. Thirty one (31) patients completed the study out of the thirty six enrolled with one subject discontinuing after the second visit, while the remaining four subjects dropped out after third visit. The male to female ratio of subjects completing all visits were 14:17 with the three male and two females dropouts citing personal reasons for opting out of the study. The end analysis revealing that 4 out of 5 dropped out subjects received placebo. All enrolled subjects had no abnormal medical history, except for gastro-intestinal disorder. Ten subjects (27.78 %) had earlier GI related medical history which had no interference with IBS. Considering the last observation carries forward method, the data of thirty five subjects (17 placebo + 18 active) was considered for efficacy analysis. The data for safety analysis was on thirty one subjects (14 placebo + 17 active). At baseline visit (Day 0), no significant difference was observed between the two treatment groups in subject demographics (Table 1). The trial was concluded after the target sample size completed their respective study procedures. All the safety and efficacy assessments were done at visits, as per schedule of events (Table 2).

**Table 1:** Subject demographics

	Placebo	Active
N (number of subjects)	18	18
Age (years)	35.4 ± 10.75	36.2 ± 11.07
Height (cm)	164.4 ± 7.71	163.1 ± 7.63
Weight (kg)	65.5 ± 10.07	65.7 ± 10.15
Body Mass Index (kg m^−2^)	24.1 ± 3.98	24.6 ± 3.14
Gender [*n* (%)]		
Male	10 (55.56)	7 (38.89)
Female	8 (44.44)	11 (61.11)

Values expressed as mean ± S.D

**Table 2:** Schedule of events

Procedures	Screening	Visit 1	Visit 2	Visit 3	Visit 4	Follow up
		(Day 0) Baseline	(Day 30)	(Day 60)	(Day 90) Final visit	(Atleast 15 days from last visit)
Informed consent	X					
Medical history	X					
Physical examination	X	X	X	X	X	
Demographics^a^		X	X	X	X	
Vital signs^b^	X	X	X	X	X	
Hematology	X				X	
Serum chemistry	X				X	
Stool test for consistency	X				X	
Urine pregnancy test^c^	X					
Randomization		X				
IP dispensing and dosing		X	X	X		
VAS assessment		X	X	X	X	
Gastrointestinal discomfort questionnaire		X	X	X	X	
Bristol stool score		X	X	X	X	
Physician’s global assessment		X	X	X		
Irritable bowel syndrome (IBS) quality of life questionnaire		X	X	X	X	
Adverse events (AEs)		X	X	X	X	X
Concomitant medications		X	X	X	X	X

^a^Age, gender, height, weight and BMI

^b^Vitals – pulse, temperature, blood pressure, heart rate, respiratory rate

^c^Urine pregnancy test at screening and on early termination

### Safety

No statistically significant changes were observed in laboratory parameters as well as the vital sign (Table 3) between the treatment groups and from the baseline to final visits. In investigator’s opinion the single AE reported during the study period was ‘unrelated’ to the study product. No serious adverse events or significant adverse events were noticed in this study.

**Table 3:** Biochemistry and haematology values between two treatment groups

Lab parameter (Units)	Visit	Placebo	Active	Normal range
Alanine aminotransferase (IU L^−1^)	Baseline	23.5 ± 6.44	22.8 ± 5.64	0 to 41
	Final Visit	23.4 ± 4.81	28.4 ± 11.20	
Albumin (g dL^−1^)	Baseline	4.2 ± 0.36	4.2 ± 0.70	3.5 to 5.2
	Final Visit	4.4 ± 0.28	4.1 ± 0.45	
Alkaline phosphatase (U L^−1^)	Baseline	76.6 ± 8.73	86.0 ± 5.98	53 to 128
	Final Visit	75.9 ± 8.76	82.8 ± 3.44	
Aspartate aminotransferase (IU L^−1^)	Baseline	24.9 ± 5.79	25.1 ± 5.63	0 to 40
	Final Visit	24.9 ± 6.56	27.4 ± 8.47	
Blood urea nitrogen (mg dL^−1^)	Baseline	12.3 ± 3.38	10.7 ± 1.66	5.0 to 24
	Final Visit	11.1 ± 2.69	10.9 ± 1.85	
Fasting blood sugar (mg dL^−1^)	Baseline	99.1 ± 2.54	106.2 ± 3.89	70 to 110
	Final Visit	104.2 ± 2.99	120.4 ± 9.48	
LDL Cholesterol (mg dL^−1^)	Baseline	105.4 ± 5.08	109.2 ± 5.69	Up to 140
	Final Visit	110.1 ± 4.30	91.3 ± 3.92	
Potassium (mEq L^−1^)	Baseline	4.1 ± 0.19	4.1 ± 0.33	3.5 to 5.2
	Final Visit	4.2 ± 0.18	4.1 ± 0.34	
Serum creatinine (mg %)	Baseline	0.9 ± 0.12	0.9 ± 0.13	0.6 to 1.4
	Final Visit	0.9 ± 0.09	0.9 ± 0.15	
Sodium (mEq L^−1^)	Baseline	139.8 ± 1.76	139.3 ± 3.30	136 to 145
	Final Visit	140.7 ± 2.58	139.6 ± 4.36	
Total bilirubin (mg dL^−1^)	Baseline	1.2 ± 1.54	0.8 ± 0.27	0.1 to 1.2
	Final Visit	0.8 ± 0.23	0.8 ± 0.23	
Total protein (g dL^−1^)	Baseline	7.1 ± 0.56	7.2 ± 0.34	6.22 to 8.0
	Final Visit	7.5 ± 0.31	7.3 ± 0.50	
Erythrocyte Count (*10^6^ cells)	Baseline	4.9 ± 0.78	5.0 ± 0.53	4.0 to 6.5
	Final Visit	4.5 ± 0.70	4.6 ± 0.61	
Haematocrit (%)	Baseline	40 ± 0.06	40 ± 0.05	40 to 50
	Final Visit	40 ± 0.06	40 ± 0.06	
Haemoglobin (gm %)	Baseline	12.8 ± 2.77	12.9 ± 1.87	11 to 16
	Final Visit	13.8 ± 2.31	13.0 ± 2.39	
Leukocyte Count (Cells cu. mm^−1^)	Baseline	6383.3 ± 16.92	6309.4 ± 16.32	4000 to 11,000
	Final Visit	7328.6 ± 18.95	6788.2 ± 16.74	
Lymphocytes (%)	Baseline	30 ± 0.06	31 ± 0.06	25 to 40
	Final Visit	30 ± 0.06	30 ± 0.06	
Monocytes (%)	Baseline	0.0 ± 0.01	0.0 ± 0.02	0 to 10
	Final Visit	0.0 ± 0.01	0.0 ± 0.01	
Neutrophils (%)	Baseline	60 ± 0.05	60 ± 0.06	40 to 75
	Final Visit	60 ± 0.06	60 ± 0.07	
Basophils (%)	Baseline	0.0 ± 0.00	0.0 ± 0.00	0 to 1
	Final Visit	0.0 ± 0.00	0.0 ± 0.00	
Eosinpophils (%)	Baseline	0.0 ± 0.01	0.0 ± 0.01	0 to 7
	Final Visit	0.0 ± 0.01	0.0 ± 0.01	
Platelet Count (*10^5^ per cu. mm)	Baseline	2.9 ± 0.67	2.7 ± 0.72	1.5 to 4.5
	Final Visit	3.2 ± 0.78	3.0 ± 0.66	

Values expressed as mean ± S.D

### Efficacy

**Fig. 2:**
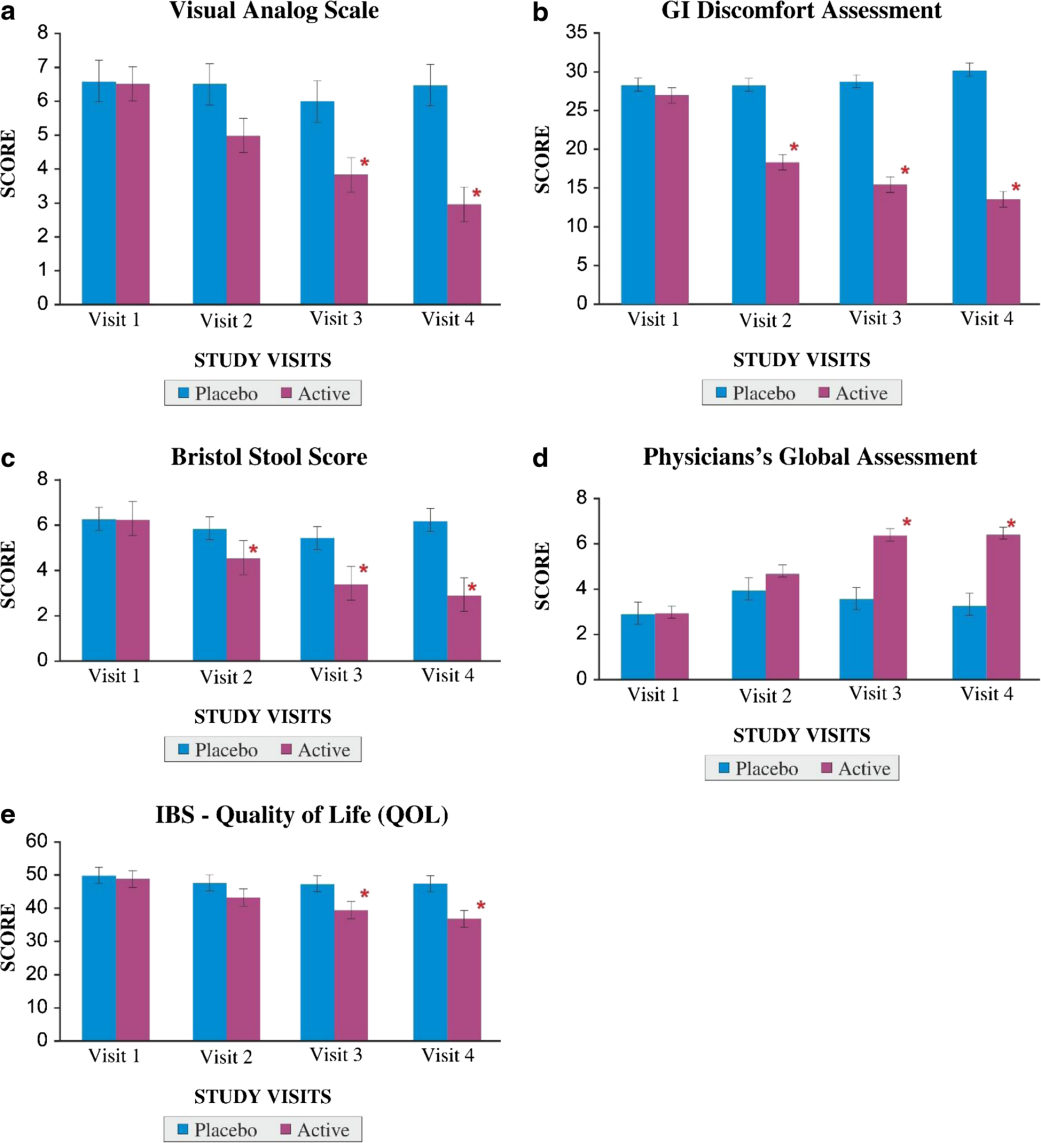
**a** Visual analog scale for abdominal pain. Legend: A value of ‘0’ indicates ‘no pain’ while ‘10’ indicates ‘worst possible pain’. *p < 0.01 between placebo and active groups on visit 3 and 4. **b** GI discomfort assessment for IBS symptoms. Legend: Low value indicates less GI discomfortness. *p < 0.01 between placebo and active groups from visits 2 to 4. **c** Bristol stool score for stool frequency. Legend: High value indicates diarrhea where the stool is ‘watery, with no solid pieces. *p < 0.01 between placebo and active groups from visits 2 to 4. **d** Physician’s global assessment for disease severity. Legend: Scale ranges from 0 to 10, where 0 indicates ‘very poor’ and 10 indicates ‘excellent’. *p < 0.01 between placebo and active groups on visits 3 and 4. **e** IBS –QOL. Legend: High QOL value indicates poor quality of life. *p < 0.01 between placebo and active groups on visits 3 and 4. All the values are expressed as mean ± S.E

As bloating, vomiting, diarrhea, abdominal pain and stool frequency are common clinical symptoms of IBS, change in these trends (which were part of GI discomfort questionnaire), throughout the study period was analyzed as primary efficacy measures. The ‘*p*’ value suggests that there was a statistically significant change in these symptoms from baseline to final visits, between the placebo and active arms. This implies that patients who received active had a significant change/decrease in clinical symptoms like bloating, vomiting, diarrhea, abdominal pain and stool frequency (Table 4) whereas the placebo arm did not exhibit any such improvement. Furthermore, assessments like VAS score for abdominal pain (Fig. 2a), GI discomfort assessment score for IBS symptoms (Fig. 2b), Bristol stool score for stool frequency (Fig. 2c), Physician's global assessment score for disease severity (Fig. 2d), IBS QOL assessment score (Fig. 2e) were found to be statistically significant (*p* < 0.01) when compared between placebo and active groups. The change in the efficacy assessments was significant (*p* < 0.01) between the two treatment groups when the respective values of their fourth visit were analyzed.

**Table 4:** Efficacy measures between two treatments from GI Discomfort Questionnaire

Parameter	Visit	Placebo	Active	*P* value
Bloating	Baseline	5.31 ± 0.82	5.88 ± 0.93	0.1372
	Final Visit	5.93 ± 0.21	3.42 ± 0.31	0.0037*
Vomiting	Baseline	4.88 ± 0.93	4.02 ± 0.55	0.1126
	Final Visit	4.42 ± 0.36	2.13 ± 0.19	0.0013*
Diarrhea	Baseline	5.79 ± 0.95	5.70 ± 0.76	0.1684
	Final Visit	5.93 ± 0.54	3.25 ± 0.42	0.0026*
Stool frequency	Baseline	5.41 ± 0.68	5.67 ± 0.58	0.1485
	Final Visit	5.75 ± 0.11	3.11 ± 0.27	0.0031*
Abdominal pain	Baseline	5.64 ± 0.77	5.71 ± 0.11	0.1539
	Final Visit	5.93 ± 0.21	1.82 ± 0.49	0.0001*

Values expressed as mean ± S.E

*Statistically significant

## Discussion

Irritable bowel syndrome (IBS), a common functional gastrointestinal (GI) disorder, is characterized by abdominal pain or discomfort, diarrhea, constipation, abdominal bloating and flatulence, which are associated with changes in the frequency and form of stool and may markedly lower the quality of life [21, 22]. Probiotics are the live microorganisms which when administered in adequate amounts confer a health benefit on the host [23]. Recently, probiotics have attracted lot of attention due to their various health benefits to humans [24, 25]. A meta-analysis of 16 randomized controlled trials evaluating the efficacy, safety, and tolerability of probiotics in IBS patients concluded that the *B. infantis* 35624 was the only probiotic which demonstrated significant benefit in the IBS patients [26]. There is significant progress on probiotics in the past decade for their diverse therapeutic efficacy [27–30]. However, *B. coagulans* MTCC 5856 strain has not been fully explored for its therapeutic efficacy in various clinical conditions. The current study was designed to evaluate the safety and efficacy of *B. coagulans* MTCC 5856 at dose of 2 × 10^9^ cfu/day in diarrhea predominant IBS patients along with standard care of treatment (Domperidone 30 mg + Esomeprazole 40 mg and Metronidazole 400 mg). In the present study, patients with diarrhea predominant IBS who received *B. coagulans* MTCC 5856 experienced statistically significant improvement from baseline in the clinical symptoms like bloating (*p* = 0.0037), vomiting (*p* = 0.0013), diarrhea (*p* = 0.0026) and stool frequency (*p* =0.0031), abdominal pain (*p* = 0.0001) vs placebo. Further, administration of *B. coagulans* MTCC 5856 resulted significant improvement in Physician's global assessment score for disease severity and IBS quality of life assessment score. The data of the study suggested that the *B. coagulans* MTCC 5856 may have potential in the adjunctive therapy for diarrhea predominant IBS, as probiotics have a beneficial effect on intestinal mucosa via several proposed mechanisms that include suppression of the growth and binding of pathogenic bacteria, improvement of the barrier function of the epithelium, and alteration of the immune activity of the host [31]. Recently concluded animal study also revealed that *B. coagulans* MTCC 5856 elicited anti-diarrhoeal activity and inhibited the gastrointestinal motility in fasted Rats [32].

When standard treatment is not completely effective, add on study design has the advantage of providing evidence of improved clinical outcomes [33]. Therefore, in the current study along with standard care of treatment (Domperidone 30 mg + Esomeprazole 40 mg and Metronidazole 400 mg), the effect of *B. coagulans* MTCC 5856 supplementation was studied in diarrhea predominant IBS patients. However, this standard care of treatment is limited to Indian sub-population. Notwithstanding, it should be noted that the probiotic, *B. coagulans* MTCC 5856, may prove its efficacy for diarrhea predominant IBS along with other standard treatments of care prevalent across the globe. Probiotics are known to produce the short chain fatty acids, an action that results in decreased luminal pH and production of bactericidal proteins [34]. Butyric acid, a by-product of bacterial fermentation of fiber, has been shown to nourish colonic enterocytes, enhancing mucosal integrity [35, 36]. Majeed et al. [37] also reported that *B. coagulans* MTCC 5856 produced short chain fatty acids (acetate, butyrate, and propionate) by fermenting plant based fibers (*Trigonella foenum-graecum, Lycium barbarum, Linum usitatissimum, Cocos nucifera, Zingiber officinale, Emblica officinalis, Plantago ovate* and *Vaccinium oxycoccos*). Despite availability of ample data, the precise mechanism of action by the probiotic in IBS and other gastrointestinal disorders still remains to be confirmed. Specific emphasis was given on the safety of *B. coagulans* MTCC 5856 in patients with diarrhea predominant IBS. The safety data of the study concluded that no significant difference in the laboratory parameters, anthropometric and vital signs from the baseline to the end of the study when supplemented with *B. coagulans* MTCC 5856 at a dose of 2 × 10^9^ cfu/day. For the first time, we report the safety and efficacy of *B. coagulans* MTCC 5856 at a dose of 2 × 10^9^ cfu/day in diarrhea predominant IBS patients along with standard care of treatment.

## Conclusions

The current study concluded that the IBS patients who received *B. coagulans* MTCC 5856 at a dose of 2 × 10^9^ cfu/day reported a significant decrease in their clinical symptoms like bloating, vomiting, diarrhea, abdominal pain and stool frequency from the placebo arm, despite both groups were on standard treatment of care. *B. coagulans* MTCC 5856 receiving patients demonstrated significant efficacy (*p* < 0.01) towards the management of IBS when compared to placebo receiving patients. All laboratory parameters, vital signs and anthropometric measurements were within the normal range during the 90 days of supplementation and no statistical difference (*p* > 0.05) between both the treatment groups. Therefore, the study confirmed that the *B. coagulans* MTCC 5856 is safe for human consumption as a dietary supplement at a dose of 2 × 10^9^ cfu/day. *B. coagulans* MTCC 5856 also demonstrated significant efficacy for IBS patients in mitigating their clinical symptoms but the mechanism of action needs to be evaluated.

## Abbreviations

AE: Adverse Event
ANCOVA: Analysis of Covariance
GCP: Good Clinical Practice
GIT: Gastro Intestinal Tract
GRAS: Generally Recognized as Safe
HDPE: High Density Polyethylene
IBS: Irritable Bowel Syndrome
ICH: International Conference on Harmonisation
ICMR: Indian Council of Medical Research
LOCF: Last Observation Carry Forward
MTCC: Microbial Type Culture Collection
QOL: Quality of Life
SAS: Statistical Analysis Software
VAS: Visual Analog Scale
